# Isolation of Marine *Bacillus* sp. with Antagonistic and Organic-Substances-Degrading Activities and Its Potential Application as a Fish Probiotic

**DOI:** 10.3390/md16060196

**Published:** 2018-06-05

**Authors:** Shuxin Zhou, Yu Xia, Chongmiao Zhu, Weihua Chu

**Affiliations:** 1 Department of Pharmaceutical Microbiology, School of Life Science and Technology, China Pharmaceutical University, Nanjing 210009, China; 1247309@stu.cpu.edu.cn; 2 Bureau of Ocean and Fisheries of Jiangsu Province, Nanjing 210003, China; xiayu66@sina.com; 3 Nanjing Zhirun Bio Technology Co., Ltd., Nanjing 211200, China; njauvet@aliyun.com

**Keywords:** *Bacillus* sp., probiotic, antagonist, multienzymes, organic substances degrading

## Abstract

We report on the isolation and characterization of an acid- and bile-tolerant bacterial strain, *Bacillus* sp. YB1701 with antibacterial and quorum-quenching activity. Strain YB1701 was isolated from coastal sediment samples and characterized by biochemical tests and 16S rRNA sequencing. In vitro study indicated that strain YB1701 can survive at pH 2.0 for up to 3 h and tolerate bile up to 2.0% concentration even after 12 h of exposure. Strain YB1701 showed antimicrobial activity against fish pathogens *Aeromonas hydrophila* and *Vibrio parahemolyticus* using an agar well diffusion assay. The trial test showed dietary supplementation of YB1701 significantly improved the resistance of *Carassius auratus gibelio* against *A. hydrophila* challenge. The safety assessment revealed that the isolate *Bacillus* sp. YB1701 was not cytotoxic to *Carassius auratus gibelio* or mice and did not exhibit hemolytic activity on rabbit blood agar plate. Disc-diffusion assays using a panel of antibiotics listed by the European Food Safety Authority (EFSA) showed that YB1701 was susceptible to selected antibiotics. Under laboratory conditions, the degradation rate of organic waste (predominately fish excrement) for 14 days by YB1701 was 79.69%. Results from the present study suggest that strain YB1701 is a potential probiotic strain and can be used in aquaculture for degrading organic waste and improving disease resistance of fish against bacterial infection. Further study is needed to assess the utility of strain YB1701 on a commercial scale.

## 1. Introduction

Over the past four decades, the aquaculture industry has been developing rapidly in China with the production accounting for more than 70% of the whole world’s output [1]. Intensification of fish farm technologies has shown high potential in improving fish production and supplying more animal protein food. However, as fish farming intensifies, the amount of bait per unit area increases, while the organic pollution caused by the farming system becomes an acute issue. Antibiotics have been used as traditional disease control strategy to combat bacteria diseases. However, the usage of antibiotics causes drug residues and the spread of antibiotic-resistance [2]. For a sustainable development of the aquaculture industry, novel strategies to control organic pollutants and bacterial infections are needed. Many studies have shown that the usage of probiotics as an alternative instead of antibiotics for *sustainable* aquaculture [3,4].

Probiotics are live microorganisms that confer a health benefit to the host when administered in adequate amounts [5,6]. Probiotics can enhance the stress tolerance and immune response, as well as improve feed digestion and pond water quality in aquaculture. The mechanisms of the probiotics include the production of antagonistic compounds that are inhibitory toward pathogens, interruption of bacteria-bacteria communication, competition with bacterial pathogen for essential nutrients, energy and attachment sites, stimulation of the host’s innate immune system, and improvement of water quality by degrading or absorbing the waste [7,8,9]. Several bacteria such as *Lactobacillus* sp. and *Bacillus* sp. can produce antimicrobial substances against fish pathogens and stimulate fish’s innate immune response [2,10]. It has been reported that *Bacillus subtilis* E20 can produce effective antimicrobial peptides against *Vibrio alginolyticus* and *V. parahaemolyticus* [11]. *Lactococcus lactis* strain A5 can producing a 3.4 kDa bacteriocin against *Bacillus cereus*, *Staphylococcus aureus* and *Salmonella thyphimurium*. *Bacillus amyloliquefaciens* FPTB16 can enhance the immunity of *Catla* [12]. Thus, the present study was carried out to isolate bacterial strains from the sediment of intertidal zone and fishing pounds with antagonistic, quorum-quenching ability and multienzymatic activities which can inhibit the quorum sensing of fish pathogen and degrade the organic pollutants in aquaculture.

## 2. Results

### 2.1. Isolation of Marine Antagonistic, Quorum-Quenching and Multienzymes Producing Strains

**Figure 1:**
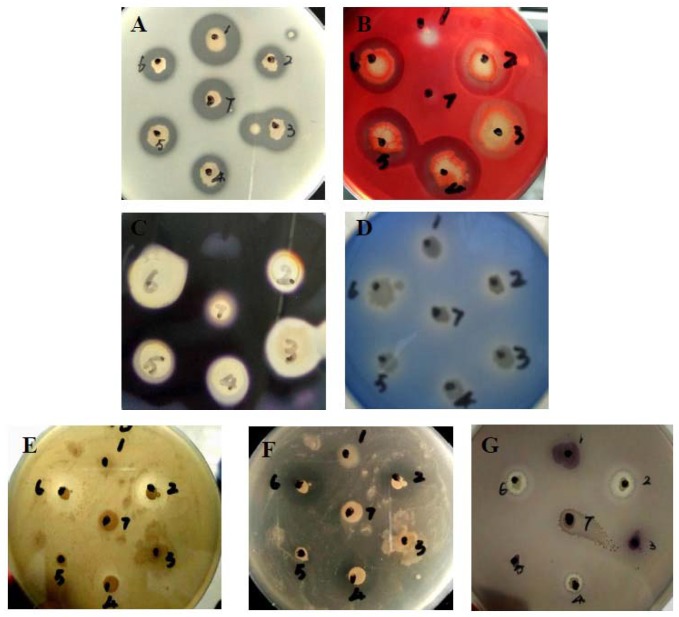
Antagonistic, quorum-quenching and extracellular enzymatic activities of selected isolates (NO. 1–7). (**A**). Proteolytic activity, (**B**). Cellulolytic activity, (**C**). Amylolytic activity, (**D**). Lipolytic activity, (**E**). Antibacterial activity against *A. hydropila* YJ-1, (**F**). Antibacterial activity against *Vibrio parahemolyticus* DX-1, (**G**). Quorum-quenching activity against *C. violaceum* CV12472.

A total of 45 bacteria were isolated and purified from the coastal sediment samples along the South Yellow Sea in Jiangsu Province, China. Seven of the isolates (NO. 1–7) with proteolysis activities on LB agar medium with 1% (*w/v*) skim milk were used for the selection of other organic hydrolases and quorum-quenching enzymes. Only the isolates NO. 2 and NO. 6, named YB1701 and YB1706, were found to exhibit strong antagonistic activity to *A. hydrophila* strain YJ-1 and *V. parahemolyticus* DX-1, with the inhibitory zones at 12.0 mm, 17.0 mm and 13.0 mm, and a 16.0 mm inhibitory diameter, respectively. YB1701 and YB1706 also can produce quorum-quenching enzyme, amylase, cellulase and lipase (Figure 1). YB1701 is a *spore*-forming bacterium resistant to heat, so it was chosen for further study.

### 2.2. Characterization and Identification of YB1701

The morphological and physiological characteristics of the strain YB1701 were compared with the data from Bergey’s Manual of Determinative Bacteriology, and strain YB1701 is a facultative anaerobe, motile, and has Gram-positive rods. The strain was positive in oxidase activity, catalase activity, gelatin liquefaction, starch hydrolysis and the citrate test. The results of biochemical tests of the strain YB1701 are shown in Table 1. 

**Figure 2:**
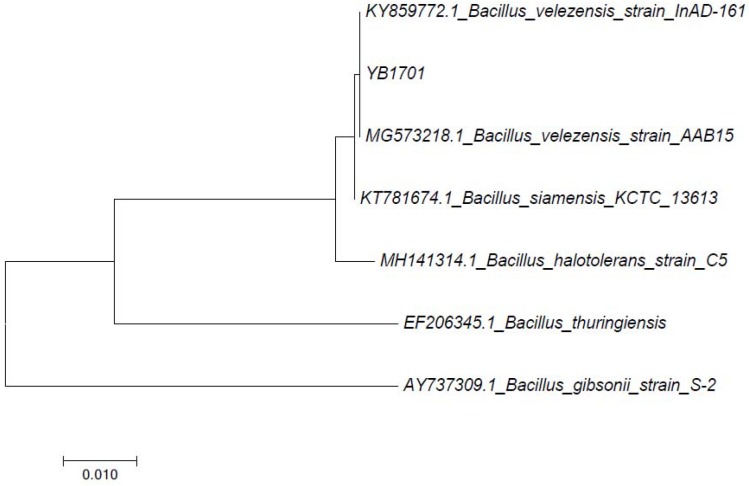
The phylogenetic tree of 16S rRNA gene sequences for *Bacillus* sp. YB1701 and other closely related *Bacillus* bacteria. The tree was generated using Neighbor-joining with scale bar equals 0.01 substitutions per nucleotide.

The partial 16S rDNA sequences of YB1701 were submitted to GenBank and the accession number was assigned as MG760246.1. Followed by BLAST analysis, the partial 16S rRNA gene sequence of YB1701 showed 100% similarity to various members of the *Bacillus* genus (Figure 2), such as *B. velezensis, B. subtilis* and *B. amyloliquefaciens* strains deposited in the NCBI database and top BLAST hit with *B. velezensis* strain NJN-6. Based on the 16S rRNA sequence and biochemical and morphological characteristics, the isolate YB1701 was identified as *Bacillus* sp. Strain YB1701 was deposited in the China General Microbiological Culture Collection Center (CGMCC, Beijing) as CGMCC No. 15605.

### 2.3. Acid and Bile Salt Tolerance

An acid and bile salt tolerance assay showed that strain YB1701 has a strong tolerance ability to acid and bile. YB1701 showed a more than 85.7% survival rate in LB broth at pH 2.0 for 3 h, and showed profound resistance to bile salt with an 85.3% survival rate in 2.0% bile for 12 h (Table 2). 

### 2.4. Safety Assessment of YB1701

Strain YB1701 has no hemolytic activity and also lacks the zone formation on the rabbit blood agar (RBA) plates (data not shown). In addition, neither mortality nor clinical symptoms of disease were observed in the tested fish treated with 0.1 mL bacterial suspension containing 10^5^–10^11^ CFU/mL of YB1701 for 7 days (data not shown). The LD_50_ value of YB1701 was estimated to exceed 10^10^ CFU for fish. Different doses of YB1701 were administrated intraperitoneally and orally to the BALB/c mice. There were no bacterial treatment-related deaths, even in groups of animals intraperitoneally treated at the highest doses. Thus, the LD_50_ for IP administrated YB1701 was more than 5 × 10^9^ CFU, and the oral LD50 for the tested strains is more than 5 × 10^10^ CFU.

### 2.5. Antibiotic Susceptibility

YB1701 was evaluated for its resistance to a panel of antibiotics, including those highlighted by the European Food Safety Authority (EFSA 2012) and recommended by the NCCLS (1997). The antibiotic resistance profile of YB1701 is listed in Table 3 which indicates that YB1701 is sensitive to all selected antibiotics as suggested by EFSA.

### 2.6. Ability of YB1701 to Degrade Organic Pollutants 

**Figure 3:**
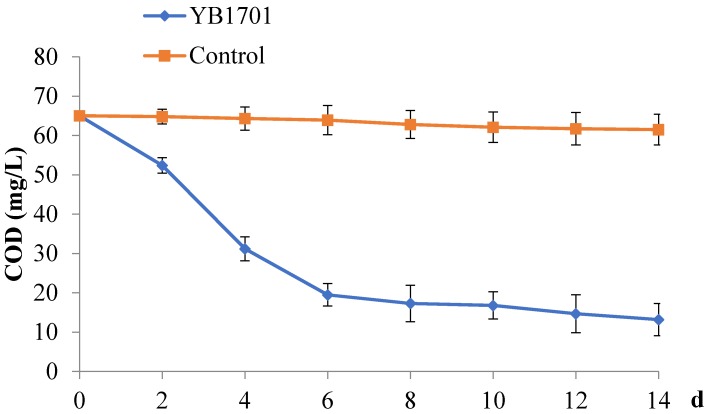
Chemical oxygen demand (COD) concentration changes in wastewater for 14 days. Error bars in Figure 3 and Figure 4 represent standard deviation of the mean (SD) from three replicate experiments (*n* = 3).

The effects of YB1701 on the degradation of organic compounds in laboratory conditions are shown in Figure 3. The removal rate of Chemical oxygen demand (COD) was above 79.69% on day 14 in YB1701 group. The value of COD in YB1701group declined quickly and reached 19.5 ± 2.84 mg/L in the first 6 days, and after that, the COD value declined steadily, and reached 13.2 ± 4.08 mg/L on day 14. The COD removal rate by YB1701 was significantly higher than the control group (*p* < 0.05).

### 2.7. In Vivo Protective Effect of YB1701 on A. hydrophila Infection

**Figure 4:**
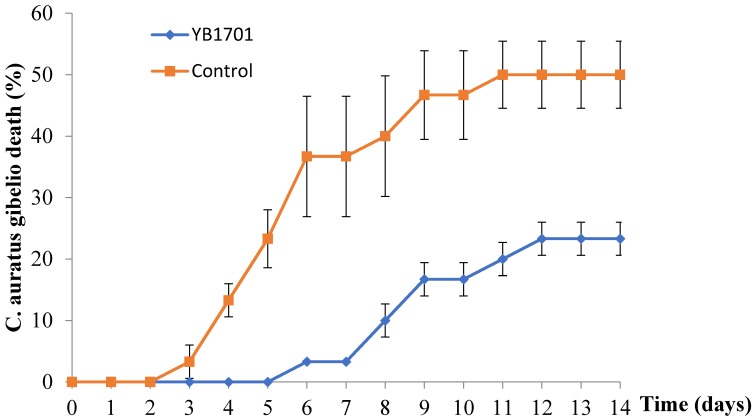
Protective effect of strain YB1701 on *C. auratus gibelio* under *A. hydrophila* challenge trial. YB1701 was administered by feeding at the concentration of 5.0 × 10^6^ CFU/g feed.

The protective effect of the diet supplemented with YB1701 on *C. auratus gibelio* against *A. hydrophila* infection was assessed, shown in Figure 4. The results indicate that YB1701 is essential in prevention of mortality of *C. auratus gibelio* by *A. hydrophila. The* cumulative mortality of the YB1701 group was only 23.3%, while that of the control group was 50%. Infection of *A. hydrophila* was observed in all dead fishes and was determined by bacterial isolation and API identification kits (data not shown).

## 3. Discussion

Alternative strategies for disease control and enhancement of water quality are important for sustainable aquaculture. The application of probiotics in aquaculture, has many advantages in contrast to antibiotics and/or chemicals for reducing disease and improving water quality [13,14]. The common microorganisms used as probiotic candidates in aquaculture are lactic acid bacteria, including the genus, *Bifidobacterium* [7,15]. Other potential probiotic candidates include the bacterial genera *Bacillus, Carnobacterium*, *Enterococcus*, *Vibrio*, *Pseudomonas* and *Streptomyces*, as well as some fungi, yeasts, and algae of the genera *Debaryomyces*, *Saccharomyces*, and *Tetraselmis* [8,16].

In this study, the potential probiotic strain *Bacillus* sp. YB1701, isolated from the coastal sediment sample, displayed positive probiotic effects in vitro including antibacterial activity against fish pathogens (*A. hydrophila* and *V. parahemolyticus*), absence of haemolysin, production of hydrolase (protease, cellulase, amylase, and lipase) and antibiotic susceptibility to all the antibiotics listed by the EFSA. Strain *Bacillus* sp. YB1701 has tested as relatively safe for in vivo application as the experimental animals (fish and mice) did not present any signs of infection or any other abnormalities after administration of *Bacillus* sp. YB1701. Our results are in concordance with other similar studies showing that *Bacillus* species are safe in animal models [17,18].

Disease resistance is an important criterion for probiotic candidate selection. Some species of *Bacillus* sp. can produce antimicrobial compounds and quorum-quenching enzymes [19,20]. The selected strain YB1701 has antagonistic activity to *A. hydrophila* and *V. parahemolyticus*, and it also produces quorum-quenching enzymes. Bacterial tests were conducted after dietary supplementation of YB1701, and the results showed that the selected strain provided significant protection to *C. auratus gibelio*. Some previous studies have also shown that some *Bacillus* sp. isolates can protect fish against bacterial infection. Gao et al. found that the marine probiotic strain, *Bacillus velezensis* V4 with anti-*A. salmonicida* properties is an effective probiotic in *Oncorhynchus mykiss* [21]. Gobi et al. observed that dietary supplementation of *Bacillus licheniformis* Dahb1 can significantly enhance the resistance against *A. hydrophila* in tilapia (*Oreochromis mossambicus*) [22]. Ran et al. found that *Bacillus* strains isolated from the intestines of catfish showed antagonism against *Edwardsiella ictaluri* and *A. hydrophila*, and showed protective effects against *E. ictaluri* in striped catfish [23]. Some other species of *Bacillus*, such as *B. aerius* [24], *B. aerophilus* [25], *B. siamensis* [26], *B. pumilus* [27], and *B. subtilis* [28], can also improve the resistance of the host against fish pathogens. *Bacillus* sp. can produce *antibacterial *compounds** such as bacteriocins (e.g., Subtilin and Coagulin) and antibiotics (e.g., Surfactin and Bacilysin). Besides the production of antimicrobial compounds can increase the disease resistance of the host, probiotics also can enhance the host’s immune response [29]. We will investigate the regulation effect of YB1701 on host immune response and characterize the bioactive compound(s) in the future.

Organic compounds affect the quality of the water and also increase the spread of bacteria and diseases. Several researchers have reported that probiotics can be used as an eco-friendly bio-control or bioremediation agents. Among the strains selected for probiotics used in aquaculture, *Bacillus* sp. is the most used genus [10]. *Bacillus* sp. can produce versatile metabolites including enzymes that are important for water quality [20,30]. The strain YB1701 that was isolated from the costal sediment can produce extracellular enzymes such as protease, amylase, cellulose, and lipase, and the strain have high ability of degrading organic waste in aquaculture water under laboratory conditions. Zhang et al. isolated two strains from the sediment of sea cucumber *Apostichopus japonicus* ponds, and the degradation rates on sediment COD were 46.95% ± 1.58% in the L15 strain and 44.31% ± 1.44% in the E4 strain [31]. Al-Wasify et al., isolated bacteria and fungi for removal of organics at the rate of 78.7% and 74.7% biological oxygen demand (BOD) removal [32]. A low-temperature organic-pollutant-degrading *Paracoccus* sp. DR8 was isolated that can degrade the feed over 5 days with COD removal rates of 50.0% [33]. The degradation rate of COD by YB1701 was 79.69% for 14 days, whereas on organic compounds, its rate was higher, which could be attributed to the test substrate being different. The composition of organic substances from feeds may be more favorable for bacterial use compared to that of pond sediment. The results indicate that YB1701 can be used as a probiotic to enhance the water quality.

## 4. Materials and Methods

### 4.1. Sample Collection and Isolation of Bacteria

Ten different sediment samples were collected from the exposed intertidal zone and fishing ponds along the South Yellow Sea in Jiangsu Province, China. Each sediment sample (approximately 100 g) was placed in a sterile plastic bag with ice bag and transported to our laboratory within 10 h and then processed immediately to isolate the bacteria. Ten grams of each sample (wet mass) were homogenized in 90 mL sterile 0.9% saline solution then 10-fold the serial dilution to 10^−4^ times. Then, 100 μL of each dilution was spread on LB agar plates containing 2% skim milk to select the microorganisms producing extracellular protease. All the plates were incubated at 28 °C for 24–48 h to determine the morphology of the colony.

### 4.2. Antimicrobial, Quorum-Quenching and Extracellular Enzymatic Activity Screening

#### 4.2.1. Antimicrobial Activity Assay 

Antimicrobial activity of the selected strains against *A. hydrophila* YJ-1 [34] and *V. parahemolyticus* DX-1 [35] were tested in vitro by an agar well diffusion test [36] with minor modification. Nutrient agar medium with 0.7% (*w/v*) agar was melted and cooled to approximately 50 °C. The respective indicator microorganism was added at the final cell density of 10^5^ CFU/mL, and the medium were immediately poured to plate with agar as top layer. When the plates were solidified, the selected strains were inoculated. The inhibition zones were detected after 24 h of incubation at 28 °C.

#### 4.2.2. Bioassay of Quorum-Quenching Activity 

The AHL biosensor *Chromobacterium violaceum* CV12472 was used to detect the quorum signal activity. All isolates were inoculated as a spot on an LB agar plate and grown overnight at 37 °C. The plate was overlaid with 10 mL of LB agar (0.5% agar) that was warmed at 50 °C and contained 10^6^ CFU/mL *C. violaceum* 12,472, which produces a short-chain autoinducer, *N*-hexanoyl homoserine lactone (C6-HSL) [37]. The plate was incubated at 28 °C for 2 days to see the inhibition of purple pigment production by CV12472 *around* the isolates.

#### 4.2.3. Extracellular Enzymatic Activities Assay

The hydrolytic extracellular enzymatic activities of potential probiotics were further detected by using the agar diffusion method. LB agar, as the experimental medium, was added to different substrates according to Nair et al. [38] with minor modifications, including skim milk (1%) for proteolytic activity, soluble starch (2%) for amylolytic activity, carboxymethyl cellulose sodium salt (*CMC-Na, 2%*) for cellulolytic activity, and Spirit Blue Agar (SBA) (Hi-Media, Mumbai, India) for lipase activity respectively. The hydrolytic enzymatic activity of the isolates inoculated on the plates were observed after incubating at 28 °C for 24 h. Proteolytic activity and lipolytic activity were determined by the transparent circle of milk degradation and the halo formation of precipitated fatty acids around the colony. To detect the amylolytic activity, after incubation at 28 °C for 24 h, the plates were flooded with 1% iodine solution at room temperature and a transparent circle around the colony indicated positivity. For cellulose detection, after incubation at 28 °C for 24 h, the plate was overlaid with Congo-red solution (0.1%) for 10 min and then washed with 1 M NaOH solution for destaining. Cellulolytic strains lacked the formation of red color around the strain. 

### 4.3. Morphological and Physiological Characteristics and 16S rRNA Gene Amplification of Potential Probiotic Strain

As for the pellet granulating process and long time storage, heat-resistant ability was selected *as* *a* *criterion*. Strain NO. 2 named YB1701 can form spores, so it was chosen for further study. The morphological (Gram staining), cultural (colony), and physiological (biochemical test) characteristics were tested. Physiological identification was undertaken as described by Bergey’s Manual of Systematic Bacteriology [39] using a trace biochemical identification reaction tube (Hangzhou BinHe Microorganism Reagent Co. Ltd., Hangzhou, China). To determine the heat resistance, the isolates were treated at 60 °C for 5 min as previously described by Dlusskaya et al. [40]. The strain with high heat-resistant ability was chosen for further study.

Genomic DNA of YB1701 was extracted using QIAamp DNA Mini Kit (QIAGEN) and quantification was done according to the described method [41]. The 16S rRNA gene of YB1701 was amplified using the upstream primer (27F 5′-AGAGTTTGATCMTGGCTCAG-3′) and the downstream primer (1492R 5′-CGGTTACCTTGTTACGACTT-3′). Partial sequencing of 16S rDNA PCR product was carried out by Shanghai Lingen Biotechnology Co., Ltd. (Shanghai, China) These sequences were submitted to GenBank and the accession number was assigned as MG760246. Multiple sequence alignment was done and phylogenetic tree was constructed by the neighbor-joining method using MEGA (Version 5.2) software. The confidence level of each branch (1000 repeats) was tested by bootstrap analysis.

### 4.4. In Vitro Acid and Bile Salt Tolerance Assay

The acid and bile tolerance of YB1701 was determined as described by Duc et al. 2004. [42] with some modifications. Different pH solutions (2.0, 3.0, and 6.5) were prepared to stimulate gastric acidic conditions by adding reagent grade HCl (35.8%) in LB medium and supplemented with pepsin from porcine gastric mucosa (Sigma-Aldric P7000, Buchs, Switzerland) at 1 mg/mL. High bile salt solutions (1.0% and 2.0%) were prepared by dissolving bile salts (Hi-media, Mumbai, India) in LB medium. One milliliter of fresh culture containing approximately 10^7^–10^8^ CFU/mL was added to the different pH solutions as well as the bile solutions and was mixed thoroughly. Cultures were then incubated at 37 °C with agitation and aliquots were removed after 30, 60 and 90 min for acid tolerance, and after 1 and 3 h for bile salt tolerance. Serial dilution was done in sterile saline (0.9% *w/v*) and the viable count was enumerated by plating on an LB agar plate. Bacterial cell survival was calculated as follows: NA/NB × 100%, where NA = log_10_ CFU/mL after incubation and NB = log_10_ CFU/mL before incubation.

### 4.5. Safety Evaluation of YB1701

Hemolytic activity assay was carried out with brackish water RBA plates at 30 °C for 2 days. Pathogenicity of YB1701 was further assayed in *Carassius auratus gibelio* and mouse models. For 50% lethal dose (LD50) determinations, YB1701 cells were washed with physiological saline (0.9% NaCl) and then subjected to 10-fold serial dilutions using the physical saline with the concentration of 10^5^–10^11^ CFU/mL, and then 0.1 mL of each dilution was injected intraperitoneally into each fish, and intraperitoneally to mouse at the levels of 5 × 10^7^, 5 × 10^8^, 5 × 10^9^ CFU/mouse and orally at 5 × 10^7^, 5 × 10^8^, and 5 × 10^10^ CFU/mouse. Each dilution trial was performed in three replicates and each group included 10 *C. auratus gibelio* (50 ± 3 g) and 10 6–8 week male BALB/c mice. Physiological saline was used as control. During this period, the activity and behavior of each animal were recorded and dead animals were removed and the mortalities were recorded daily for 7 days. The LD_50_ value was calculated by the method of Reed and Muench [43].

### 4.6. Antibiotic Susceptibility Assay

The susceptibility of *Bacillus* sp. YB1701 was obtained by the disc diffusion method according to the method described by the National Committee for Clinical Laboratory Standards (NCCLS, 2012) [44] using antibiotic discs (Oxoid, UK). Antimicrobials required to be examined in *Bacillus* sp. are vancomycin, gentamicin, kanamycin, streptomycin, erythromycin, clindamycin, tetracycline, and chloramphenicol, as suggested by EFSA [45]. The reading was performed by measuring the diameter of the no bacteria growing zone (inhibiting zone) around the disk.

### 4.7. Degradation Rates of YB1701 for Organics in Wastewater of Aquaculture

The degradation rate of the isolate for organics removal was evaluated by chemical oxygen demand (COD). Aquaria (90 × 55 × 45 cm) with 100 L *C. auratus gibelio* culture wastewater with continuous aeration were selected for experimental tanks. The initial COD was adjusted to 65.0 mg/L using the normal feed. Culture broth of strain YB1701 containing 1 × 10^8^ CFU/mL was added to the aquaria water samples by adding a ratio of 1 × 10^5^ CFU/mL to evaluate its effect on wastewater. The experiment was divided into two groups (with and without adding YB1701), with each group containing three duplicates. 

### 4.8. Effect of YB1701 Supplemented Fish Diet on Disease Resistance

*Bacillus* sp. strain YB1701 was grown in LB medium at 37 °C for 36 h in a rotary shaker. Bacterial cells were harvested by centrifugation, washed with saline solution, and then resuspended in saline solution. The solution was then mixed with pelleted basal diet and air-dried at 50 °C in the oven for 2–5 h to obtain about 5.0 × 10^6^ CFU/g feed and kept at 4 °C for use.

Groups of 30 healthy *C. auratus gibelio* (50 ± 3 g) were fed with (at the concentration of 5.0 × 10^6^ CFU/g feed) or without YB1701 separately for 30 days. After that, fish were tested intraperitoneally with 0.1 mL of *A. hydrophila* YJ-1 suspension containing 2.0 × 10^5^ cells at the dose causing 50% mortality (LD_50_). The diet was continued for 14 days after infection. The mortality in each group was recorded daily. Dead fish were removed from the aquaria daily, and their livers and kidneys were subjected to pathogen isolation on ampicillin dextrin agar (ADA) as described by Latif-Eugenín [46]. After incubation at 30 °C for 24 h, the bacteria growth on the medium were identified by using the API 20 NE test kit.

### 4.9. Ethics Statement

All experiments conducted with vertebrate animals (carp and mice) were approved by the Animal Care and Use Committee (ACUC) review boards at China Pharmaceutical University in accordance with the respective animal welfare guidelines specified by the government of China.

## 5. Statistical Analysis

All data were analyzed by using One-way Analysis of Variance (ANOVA), and multiple comparison tests (Duncan’s and Tukey’s-tests) were performed using SPSS Statistic 2.0 software. Data were presented as Mean ± Standard. *p* < 0.05 was considered statistically significant.

## 6. Conclusions

The antagonistic and multienzymes producing bacteria *Bacillus* sp. was isolated from coastal sediment and the preliminary probiotic effects of bacterial disease resistance and degradation of organic waste water was characterized. This is the first report on the isolation of a *Bacillus* strain with both antibacterial and organic substances degrading activity. The results of this study indicate that the *Bacillus* sp. YB1701 showed potential for biological control of fish bacterial disease and degrading organic waste water in aquaculture. Further study is needed to evaluate the practical concentrations on a commercial scale.

## Figures and Tables

**Table 1:** Morphological and biochemical properties of *Bacillus* sp. YB1701.

Characteristics	*Bacillus* sp. YB1701
Morphological	
Shape	Rods
Gram stain	G^+^
Motility	Motile
Spore formation	+
Growth	
Growth temperature	15 °C–50 °C
Growth pH	5–8
Growth in 10% NaCl	+
Aerobic growth	+
Anaerobic growth	+
Biochemical tests	
Catalase	+
Oxidase	+
Voges-Proskauer	+
Indole production	-
Methyl red test	-
Citrate	+
Catalase reaction	+
Glucosamine	+
Nitrate reduction	+
H_2_S production	+
Crystalline dextrin production test	-
Fermentative	
Glucose	+
Fructose	+
Lactose	-
Mannose	-
Raffinose	+
Hydrolysis of	
Casein	+
Gelatin	+
Starch	+

Note: +: Positive; −: Negative.

**Table 2:** In vitro acid and bile tolerance of YB1701.

-	Log_10_ CFU of Viable Bacteria per mL			-	Log_10_ CFU of Viable Bacteria per mL		
Time/h	pH 6.5	pH 3.0	pH 2.0	Time/h	0.0% bile	1.0% bile	2.0% bile
0	7.98	7.98	7.98	0	8.76	8.76	8.76
1	7.89	7.63	7.25	1	8.15	8.13	7.94
2	8.14	7.18	7.18	3	7.96	7.95	7.32
3	8.27	7.03	6.84	12	7.82	7.87	7.47

**Table 3:** Antibiotics sensitivity test of YB1701.

Antibiotic Discs *	Inhibition Zone Diameter (mm) #	Susceptibility
Gentamicin	23.4 ± 0.4	S
Kanamycin	27.0 ± 0.2	S
Clindamycin	25.4 ± 1.4	S
Chloramphenicol	22.6 ± 0.6	S
Erythromycin	27.5 ± 0.5	S
Streptomycin	21.7 ± 0.7	S
Tetracycline	13.4 ± 0.6	S
Vancomycin	20.2 ± 1.1	S

* Antibiotic-impregnated discs (6 mm) with amount in μg ± SE shown in brackets; # Diameter of inhibition from three individual experiments; S, sensitive; I, intermediate; R, resistant.

## References

[1] Food and Agriculture Organization (FAO) (2012). The State of World Fisheries and Aquaculture, 2010.

[2] Watts J.E.M., Schreier H.J., Lanska L., Hale M.S. (2017). The rising tide of antimicrobial resistance in aquaculture: Sources, sinks and solutions. Mar. Drugs.

[3] Hossain M.I., Sadekuzzaman M., Ha S.D. (2017). Probiotics as potential alternative biocontrol agents in the agriculture and food industries: A review. Food Res. Int..

[4] Hai N.V. (2015). The use of probiotics in aquaculture. J. Appl. Microbiol..

[5] Hill C., Guarner F., Reid G., Gibson G.R., Merenstein D.J., Pot B., Morelli L., Canani R.B., Flint H.J., Salminen S. (2014). Expert consensus document. The International Scientific Association for Probiotics and Prebiotics consensus statement on the scope and appropriate use of the term probiotic. Nat. Rev. Gastroenterol. Hepatol..

[6] Food and Agriculture Organization (FAO)/World Health Organization (WHO) (2002). Guidelines for the Evaluation of Probiotics in Food.

[7] Verschuere L., Rombaut G., Sorgeloos P., Verstraete W. (2000). Probiotic bacteria as biological control agents in aquaculture. Microbiol. Mol. Biol. Rev..

[8] Cruz P.M., Ibáñez A.L., Hermosillo O.A.M., Saad H.C.R. (2012). Use of probiotics in aquaculture. ISRN Microbiol..

[9] Huynh T.G., Shiu Y.L., Nguyen T.P., Truong Q.P., Chen J.C., Liu C.H. (2017). Current applications, selection, and possible mechanisms of actions of synbiotics in improving the growth and health status in aquaculture: A review. Fish Shellfish Immunol..

[10] Banerjee G., Ray A.K. (2017). The advancement of probiotics research and its application in fish farming industries. Res. Vet. Sci..

[11] Cheng A.C., Lin H.L., Shiu Y.L., Tyan Y.C., Liu C.H. (2017). Isolation and characterization of antimicrobial peptides derived from *Bacillus subtilis* E20-fermented soybean meal and its use for preventing *Vibrio* infection in shrimp aquaculture. Fish Shellfish Immunol..

[12] Singh S.T., Kamilya D., Kheti B., Bordoloi B., Parhi J. (2017). Paraprobiotic preparation from *Bacillus amyloliquefaciens* FPTB16 modulates immune response and immune relevant gene expression in *Catla catla* (Hamilton, 1822). Fish Shellfish Immunol..

[13] Zorriehzahra M.J., Delshad S.T., Ade L.M., Tiwari R., Karthik K., Dhama K., Lazado C.C. (2016). Probiotics as beneficial microbes in aquaculture: An update on their multiple modes of action: A review. Vet. Q..

[14] Bentzon-Tilia M., Sonnenschein E.C., Gram L. (2016). Monitoring and managing microbes in aquaculture—Towards a sustainable industry. Microb. Biotechnol..

[15] Vázquez L., Hernández R., Sainz E., González Cervantes R., Martínez Cruz P., Mayorga Reyes L., Azaola Espinosa A. (1996). Cambio en la flora intestinal de ratones por la administraci ´on de bifidobacterias y jugo de girasol. Vet. Méx..

[16] Tan L.T., Chan K.G., Lee L.H., Goh B.H. (2016). *Streptomyces* bacteria as potential probiotics in aquaculture. Front. Microbiol..

[17] Mingmongkolchai S., Panbangred W. (2018). *Bacillus* probiotics: An alternative to antibiotics for livestock production. J. Appl. Microbiol..

[18] Elshaghabee F.M.F., Rokana N., Gulhane R.D., Sharma C., Panwar H. (2017). *Bacillus* as potential probiotics: Status, concerns, and future perspectives. Front. Microbiol..

[19] Zhao J., Chen M., Quan C.S., Fan S.D. (2015). Mechanisms of quorum sensing and strategies for quorum sensing disruption in aquaculture pathogens. J. Fish Dis..

[20] Hong H.A., Duc le H., Cutting S.M. (2005). The use of bacterial spore formers as probiotics. FEMS Microbiol. Rev..

[21] Gao X.Y., Liu Y., Miao L.L., Li E.W., Sun G.X., Liu Y., Liu Z.P. (2017). Characterization and mechanism of anti-*Aeromonas salmonicida* activity of a marine probiotic strain, *Bacillus velezensis* V4. Appl. Microbiol. Biotechnol..

[22] Gobi N., Vaseeharan B., Chen J.C., Rekha R., Vijayakumar S., Anjugam M., Iswarya A. (2018). Dietary supplementation of probiotic *Bacillus licheniformis* Dahb1 improves growth performance, mucus and serum immune parameters, antioxidant enzyme activity as well as resistance against *Aeromonas hydrophila* in tilapia *Oreochromis mossambicus*. Fish Shellfish Immunol..

[23] Ran C., Carrias A., Williams M.A., Capps N., Dan B.C.T., Newton J.C., Kloepper J.W., Ooi E.L., Browdy C.L., Terhune J.S. (2012). Identification of *Bacillus* strains for biological control of catfish pathogens. PLoS ONE.

[24] Meidong R., Khotchanalekha K., Doolgindachbaporn S., Nagasawa T., Nakao M., Sakai K., Tongpim S. (2017). Evaluation of probiotic *Bacillus aerius* B81e isolated from healthy hybrid catfish on growth, disease resistance and innate immunity of Pla-mong *Pangasius bocourti*. Fish Shellfish Immunol..

[25] Ramesh D., Souissi S., Ahamed T.S. (2017). Effects of the potential probiotics *Bacillus aerophilus* KADR3 in inducing immunity and disease resistance in *Labeo rohita*. Fish Shellfish Immunol..

[26] Meidong R., Doolgindachbaporn S., Jamjan W., Sakai K., Tashiro Y., Okugawa Y., Tongpim S. (2017). A novel probiotic *Bacillus siamensis* B44v isolated from Thai pickled vegetables (Phak-dong) for potential use as a feed supplement in aquaculture. J. Gen. Appl. Microbiol..

[27] Srisapoome P., Areechon N. (2017). Efficacy of viable *Bacillus pumilus* isolated from farmed fish on immune responses and increased disease resistance in Nile tilapia (*Oreochromis niloticus*): Laboratory and on-farm trials. Fish Shellfish Immunol..

[28] Hao K., Wu Z.Q., Li D.L., Yu X.B., Wang G.X., Ling F. (2017). Effects of dietary administration of *Shewanella xiamenensis* A-1, *Aeromonas veronii* A-7, and *Bacillus subtilis*, single or combined, on the grass Carp (*Ctenopharyngodon idella*) intestinal microbiota. Probiotics Antimicrob. Proteins.

[29] De B.C., Meena D.K., Behera B.K., Das P., Das Mohapatra P.K., Sharma A.P. (2014). Probiotics in fish and shellfish culture: Immunomodulatory and ecophysiological responses. Fish Physiol. Biochem..

[30] Mondol M.A., Shin H.J., Islam M.T. (2013). Diversity of secondary metabolites from marine *Bacillus* species: Chemistry and biological activity. Mar. Drugs.

[31] 35Zhang D.S., Li H., Liu Y., Qiao G., Chi S., Song J. (2016). Screening and identification of organics-degrading bacteria from the sediment of sea cucumber *Apostichopus japonicus* ponds. Aquacult. Int..

[32] Al-Wasify R.S., Ali M.N., Hamed S.R. (2017). Biodegradation of dairy wastewater using bacterial and fungallocal isolates. Water Sci. Technol..

[33] Yan F.J., Tian X.L., Dong S.G., Wu X.H., Liu B.G. (2012). Isolation, selection and characterization of low temperature organic-pollutant-degrading bacteria in sea cucumber ponds. J. Fish Sci. China.

[34] Chu W.H. (2001). Studies on the pathology and control of the bacterial hemorrhage disease in a hybrid crussian carp. Reserv. Fish..

[35] Chu W.H., Zhu W., Kang C.T. (2009). Isolation, identification of marine *Bdellovibrios* and its effect on *Vibrio parahaemolyticus*. Microbiology.

[36] Perez C., Paul M., Bazerque P. (1990). An antibiotic assay by the agar well diffusion method. Acta Biol. Med. Exp..

[37] McLean R.J., Pierson L.S., Fuqua C. (2004). A simple screening protocol for the identification of quorum signal antagonists. J. Microbiol. Methods.

[38] Nair A.V., Vijayan K.K., Chakrabort K., Antony M.L. (2012). Diversity and characterization of antagonistic bacteria from tropical estuarine habitats of Cochin, India for fish health management. World J. Microbiol. Biotechnol..

[39] Holt J.G., Krieg N.R., Sneath P.H.A., Staley J.T., Williams S.T. (1994). Bergey’s Manual of Determinative Bacteriology.

[40] Dlusskaya E.A., McMullen L.M., Gänzle M.G. (2011). Characterization of an extremely heat-resistant *Escherichia coli* obtained from a beef processing facility. J. Appl. Microbiol..

[41] Sambrook J., Fritch E.F., Maniatis T. (1982). Molecular Cloning: A Laboratory Manual.

[42] Duc L.H., Hong H.A., Barbosa T.M., Henriques A.O., Cutting S.M. (2004). Characterization of *Bacillus* probiotics available for human use. Appl. Environ. Microbiol..

[43] Reed L.J., Muench H. (1938). A simple method of estimating fifty per cent endpoints. Am. J. Epidemiol..

[44] NCCLS (2012). Methods for Dilution Antimicrobial Susceptibility Tests for Bacteria that Grow Aerobically, Approved Standard.

[45] EFSA (2012). Guidance on the assessment of bacterial susceptibility to antimicrobials of human and veterinary importance. EFSA J..

[46] Latif-Eugenín F., Beaz-Hidalgo R., Figueras M.J. (2016). Evaluation of different conditions and culture media for the recovery of *Aeromonas* spp. from water and shellfish samples. J. Appl. Microbiol..

